# Correlation Analysis between Tamoxifen and Lumbar Intervertebral Disc Degeneration: A Retrospective Case-Control Study

**DOI:** 10.1155/2022/3330260

**Published:** 2022-05-31

**Authors:** Xiangyu Li, Ruoyu Zhao, Samuel Rudd, Wenyuan Ding, Sidong Yang

**Affiliations:** ^1^Department of Spine Surgery, The Third Hospital of Hebei Medical University, 139 Ziqiang Road, Shijiazhuang 050051, China; ^2^School of Chemical Engineering, The University of Queensland, St Lucia 4072, Queensland, Australia; ^3^Hebei Joint International Research Center for Spinal Diseases, 139 Ziqiang Road, Shijiazhuang 050051, China

## Abstract

**Objectives:**

To investigate the correlation between tamoxifen (TAM) and lumbar intervertebral disc (IVD) degeneration (IVDD).

**Methods:**

The patients who visited the department of spine surgery from January 2015 to December 2020 were retrospectively reviewed. Those with a history of breast cancer surgery were identified and their data were collected. These data included patients' age, body mass index (BMI), menstrual history, postoperative history, drug treatment plan, and imaging data. The participants were divided into the TAM group and the non-TAM group. Lumbar IVDD was assessed by lumbar lordosis (LL), vertebral CT density, lumbar disc height index (DHI), Modic changes, and modified Pfirrmann grading score. SPSS 20 was used for statistical analysis.

**Results:**

A total of 75 patients were included in this study, 46 patients in the TAM group and 29 patients in the non-TAM group. No significant differences were present in age, BMI, postoperative history, LL, and vertebral CT density between the two groups. The DHI of L1/2 and L2/3 in the TAM group was lower compared to the non-TAM group (*P*=0.038 and *P*=0.034, respectively), while comparisons regarding the DHI of L3/4, L4/5, and L5/S1, and the average DHI between TAM and non-TAM groups were not significant. The modified Pfirrmann grading scores of the L1/2 and L2/3 IVDs in the TAM group were higher than those in the non-TAM group (*P*=0.004 and *P*=0.025, respectively). Comparisons of L3/4, L4/5, and L5/S1 between the two groups were not significant. The comparisons regarding the occurrence of Modic changes did not show a significant difference between the TAM and non-TAM groups.

**Conclusions:**

This study indicates that there might be some positive correlation between TAM use and lumbar IVDD. In particular, the degeneration of L1/2 and L2/3 has shown a correlation with TAM use.

## 1. Introduction

Lower back pain is one of the most common and important public health problems that afflict adults, bringing significant life troubles and social and economic burdens [1, 2]. Intervertebral disc (IVD) degeneration (IVDD) is an important cause of lower back pain, but the mechanism of IVDD is still unclear. The cause of IVDD may be related to a decrease in the number of nucleus pulposus cells (NPCs) and disruption of the extracellular matrix (ECM) balance caused by age, inflammation, trauma, genetics, and other factors [3, 4]. An earlier preliminary clinical study showed that ovariectomy (OVX) resulted in a significant decrease in the estrogen level of patients; moreover, the Pfirrmann grading of the lumbar IVDs in OVX patients was higher than that in normal patients [5]. A large number of experiments have evidenced that estrogen can enhance the tolerance of NPCs to inflammation and oxidative stress, reduce cell apoptosis, and improve cell survival rate [3, 4, 6–8]. Therefore, the level of estrogen can influence the progression of IVDD.

Endocrine therapy is an important treatment for certain tumors, such as breast and ovarian cancer, which can effectively reduce tumor metastasis and improve patient survival. Tamoxifen (TAM), a selective estrogen receptor modulator, is commonly used in premenopausal estrogen receptor (ER)-positive breast cancer patients. However, a large number of studies have found that the use of TAM negatively affects the bones, uterus, and other organs [9, 10]. Previous investigations have revealed the expression of estrogen receptors in IVD tissues; so far, however, no studies have reported the effect of estrogen receptor modulators (e.g., TAM) on IVDD [4].

Thus, this study retrospectively analyzed and compared IVDD between breast cancer patients who took TAM and those without TAM, in order to investigate the correlation between TAM and IVDD.

## 2. Materials and Methods

### 2.1. Patients

The postoperative breast cancer patients who visited the department of spine surgery from January 2015 to December 2020 were retrospectively reviewed and divided into two groups based on the use of TAM: the TAM group and the non-TAM group (breast cancer patients treated without TAM after surgery). Patients in the TAM group received TAM at a dose of 10 mg/bid for at least six months.

### 2.2. Inclusion Criteria

1. Postoperative breast cancer patients without menopause took TAM or those who did not take TAM
2. Patients who have complete clinical data

### 2.3. Exclusion Criteria

1. Patients who failed to receive regular postoperative chemotherapy
2. Patients suffering from endocrine system and immune system diseases that may affect hormone levels
3. Patients with spinal trauma, spinal fracture, and history of spinal surgery

### 2.4. Data Collection and Calculations

**Figure 1:**
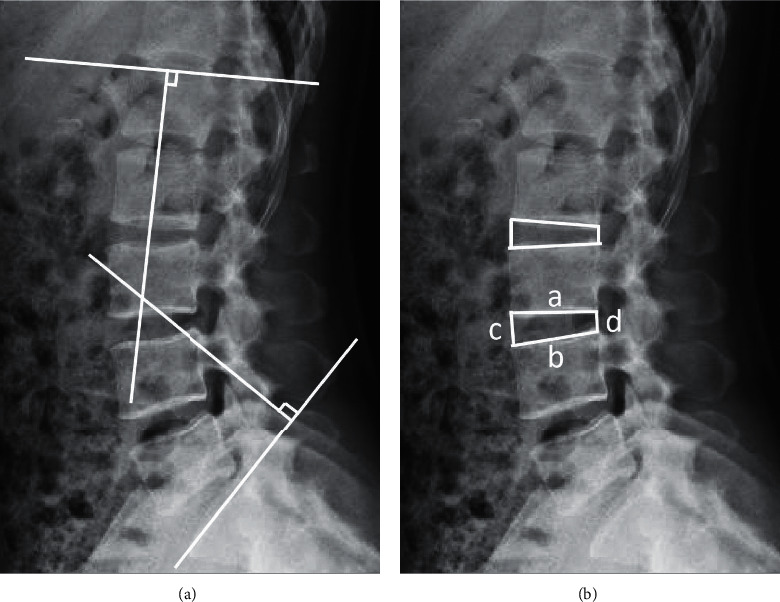
Lumbar lordosis (LL) and lumbar disc height index (DHI) measurements. (a) LL: the angle between the tangent line of the upper endplate of the L1 vertebral body and S1 vertebral body in the lateral lumbar spine. (b) DHI: (intervertebral leading-edge height + posterior edge height)/(intervertebral upper body width + lower body width) ^*∗*^ 100%, where a is the intervertebral upper body width, b is the intervertebral lower body width, c is the intervertebral anterior edge height, and d is the intervertebral posterior edge height.

The postoperative data of female breast cancer patients in the department of spinal surgery were retrospectively analyzed. The following patient data were analyzed: age, body mass index (BMI), menstrual history, postoperative history of breast cancer, and drug treatment plan. Imaging data included lumbar MRI, CT, and X-ray. Lumbar lordosis (LL) was measured by a lumbar X-ray or CT of the angle between the tangent line of the upper endplate of the L1 vertebral body and the S1 vertebral body in the lateral lumbar spine (Figure 1(a)). The lumbar disc height index (DHI) was calculated using the following formula (Figure 1(b)) [11, 12]:

(1) $$\begin{array}{c} \left(\frac{\text{intervertebral anterior edge height}+\text{posterior edge height}}{\text{intervertebral upper body width}+\text{lower body width}}\right)\times 100\%. \end{array}$$

**Figure 2:**
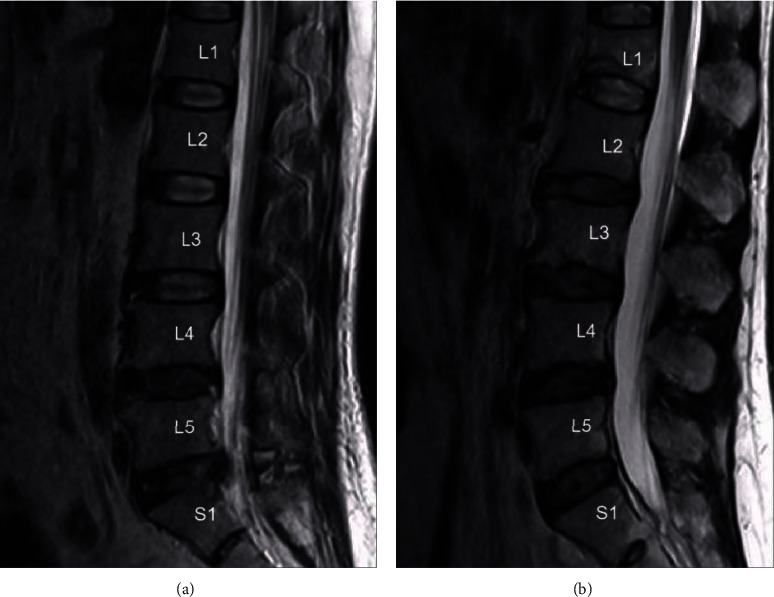
MRI T2-weighted sagittal images were used to assess the degree of IVDD at the L1/L2–L5/S1 levels by the modified Pfirrmann grading system. (a) The modified Pfirrmann grading scores from L1/2 to L5/S1 were 2, 2, 3, 4, and 4 for a patient in the non-TAM group, respectively. (b) The modified Pfirrmann grading scores from L1/2 to L5/S1 were 2, 4, 4, 4, and 4 for a patient in the TAM group, respectively.

**Figure 3:**
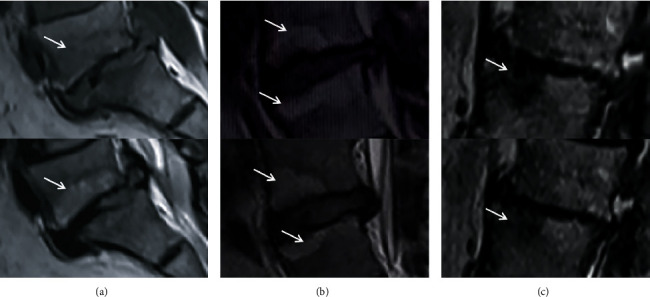
Modic classification for vertebral endplate changes (the patients pictured were not included in the study). (a) Type I: decreased signal on T1- and increased signal on T2-weighted images. (b) Type II: increased signals on both T1- and T2-weighted images. (c) Type III: decreased signals on both T1- and T2-weighted images.

MRI T2-weighted sagittal images were utilized to assess the degree of IVDD at the L1/L2–L5/S1 levels using the modified Pfirrmann grading system, as per Figure 2 and Table 1 [13, 14]. MRI T1- and T2-weighted sagittal images were used to evaluate whether Modic changes appeared in the upper and lower endplates of the vertebral body and the bone marrow, as shown in Figure 3 [15]. The average vertebral CT density of the five vertebral bodies was measured by CT. All data were collected and evaluated by two independent spinal surgeons, and significant differences were resolved by a consensus. This study was approved by the Ethics Committee of the Third Hospital of Hebei Medical University, in accordance with the provisions of the Declaration of Helsinki. Informed consent was obtained from each patient before the study, and all data remained anonymous.

### 2.5. Statistical Analysis

Statistical analysis was performed using SPSS 20 (SPSS Inc., Chicago, IL, USA). Measurement data were expressed as mean ± standard deviation (SD). The comparisons of age, postoperative history, LL, vertebral CT density, and lumbar DHI were performed by the independent sample *t*-test, whereas the comparison of the modified Pfirrmann grading score and occurrence of Modic changes were conducted by a nonparametric test. *P* < 0.05 was considered statistically significant.

## 3. Results

A total of 75 patients were enrolled in this study, including 46 patients in the TAM group and 29 patients in the non-TAM group. No significant differences were present in age, BMI, and postoperative history between the two groups. The LL of the TAM group (31.15 ± 9.26) was higher than that of the non-TAM group (30.54 ± 6.96), but there was no significant difference. In addition, there was no significant difference in vertebral CT density between the TAM group (185.21 ± 53.66) and the non-TAM group (184.23 ± 50.03), as shown in Table 2.

DHI of L1/2, L2/3, L3/4, L4/5, and L5/S1 is 24.96 ± 4.04, 27.28 ± 5.16, 30.05 ± 5.70, 32.06 ± 5.21, and 28.63 ± 6.18 in the TAM group and 26.86 ± 3.41, 29.68 ± 3.72, 31.23 ± 3.81, 31.35 ± 5.11, and 28.49 ± 7.46, respectively, in the non-TAM group (Table 3). The L1/2 and L2/3 DHI in the TAM group were lower than those in the non-TAM group (*P*=0.038 and *P*=0.034, respectively), whereas comparisons regarding the DHI of L3/4, L4/5, and L5/S1 between TAM and non-TAM groups were not significant. The average DHI of the TAM group (29.69 ± 3.08) was higher than that of the non-TAM group (28.52 ± 3.47), but there was no statistical difference, as shown in Table 3.

All the patients' IVDs from L1/2 to L5/S1 were evaluated through the modified Pfirrmann grading system. The modified Pfirrmann grading scores of the L1/2 and L2/3 IVDs in the TAM group were higher than those in the non-TAM group (*P*=0.004 and *P*=0.025, respectively), while the comparisons of L3/4, L4/5, and L5/S1 between the two groups were not significant, as shown in Table 4.

All Modic changes were Modic type II changes. The comparisons regarding the occurrence of Modic changes did not show a significant difference between the TAM and non-TAM groups, as shown in Table 5.

## 4. Discussion

IVDD is a primary cause of lower back pain. Although the etiology of IVDD is still unclear, recent studies have found that IVDD is accompanied by a decrease in the number of NPCs and disruption of the ECM balance. Furthermore, apoptosis, inflammation, and senescence of the NPCs can accelerate this process [1–4]. Anti-inflammatory and antioxidant factors have become a research hotspot in the field of IVDD. Many researchers have studied the correlation between estrogen and IVDD and found that estrogen can inhibit NPC apoptosis and delay the progression of IVDD through a variety of ways, for example, enhancing the anti-inflammatory and antioxidant capacity of NPCs, activating autophagy, promoting ECM synthesis, and inhibiting matrix metalloproteinases [3–7]. Our previous studies have shown that estrogen can inhibit apoptosis of NPCs by activating NF-*κ*B and PI3K-Akt signaling pathways, which has been confirmed in animal experiments [6–8]. Wang et al. evidenced the expression of ER in IVD tissues [4]. The clinical study by Zhao et al. also established that OVX led to a significant decrease in the female estrogen level and promoted the progression of IVDD over a long period of time [5]. Therefore, the level of estrogen can influence the progression of IVDD.

Breast cancer is one of the most common tumors in women, and approximately 70% of them are ER-positive. Endocrine therapy in these patients can effectively reduce tumor metastasis and improve survival rates. TAM is the first choice for endocrine therapy in premenopausal ER-positive patients. As a selective estrogen receptor modulator, it can competitively bind to estrogen receptors, exerting antagonistic or estrogen-like effects [16, 17]. Studies have shown that TAM can affect other tissues or organs, such as the uterus and bones. The use of TAM can lead to endometrial thickening, polyps, and endometrial cancer. Jeon et al. found that TAM can delay postmenopausal bone mineral density (BMD) loss in women, which has been confirmed by animal experiments of ovariectomy, but the mechanism of TAM's action is still unclear [9, 10].

This study retrospectively analyzed and compared vertebral CT density, LL, lumbar DHI, and modified Pfirrmann grading of breast cancer patients who took TAM and those who did not take TAM, in order to investigate the correlation between TAM and lumbar IVDD. A total of 75 patients were enrolled, 46 patients in the TAM group and 29 patients in the non-TAM group.

Age, gender, and obesity are important factors that would influence IVDD. Many studies have confirmed that increasing age and abnormal obesity can promote the progression of IVDD [18, 19]. Ekşi M. Ş. et al. found that severe IVDD was more common in women than in men, and this difference was significant at all lumbar levels except L5/S1 [18]. This may be related to the differences in body structure and hormone levels caused by the gender. There are many ways of assessing the degree of obesity in patients, and the most commonly used is BMI [19, 20]. Fat content is also one of the indicators for evaluating obesity. Berikol G et al. found that subcutaneous fat tissue thickness at the L1/L2 level was better than BMI in predicting lower back pain and IVDD [20]. In this study, there were no significant differences in age and BMI between the two groups of patients. This reduced the interference of age and obesity on the results of this study. Moreover, the comparison of postoperative medical history between the two groups was not significant.

The normal lumbar physiological curvature is essential for the maintenance of the balance of the entire spine [1, 2]. Changes in LL can affect the internal balance of the spine and accelerate lumbar degeneration. Yang et al. found that reduced LL leads to decreased spinal elasticity and mobility, which may be a risk factor for IVDD [21]. In this study, spinal injury, spinal fracture, and other factors affecting the physiological curvature of the spine were excluded. The results showed no significant difference in LL between the two groups, although both groups had a lower than normal physiological curvature in adults, indicating that TAM did not significantly affect the physiological curvature of the spine.

Vertebral CT density values were used to assess the degree of osteoporosis in the patients [11]. Estrogen has a regulatory effect on multiple organs and systems, such as the reproductive organs and bones, especially in postmenopausal women, who suffer from severe osteoporosis due to reduced hormone levels [22]. However, previous studies on the relationship between vertebral BMD and IVDD have not yielded clear and consistent results. Most scholars, such as Salo et al., speculated that increased vertebral BMD promotes IVDD, caused by reduced nutrient supply to the IVD due to increased endplate calcification [23]. However, previous studies found no significant positive or negative correlation between vertebral BMD and IVDD [24–26]. This study established no significant difference in the vertebral CT density between the two groups, suggesting that TAM as an ER modulator does not significantly affect vertebral BMD in premenopausal women, but may affect the degree of IVDD through other pathways.

In the present investigation, the level of IVDD was assessed by the lumbar DHI and the modified Pfirrmann grading system [11, 12]. Akeda K et al. observed that the DHI of the elderly would decrease significantly over a decade, accompanied by an increase in the Pfirrmann grading score [27]. Wang et al. found that postmenopausal women had higher Pfirrmann grading scores than premenopausal women, suggesting more severe IVDD [4]. Zhao et al. also found that patients with OVX had a higher lumbar disc grading score than those without ovariectomies [5]. In this study, the comparison results of the two methods showed that TAM promoted the degeneration of the upper lumbar discs (L1/2, L2/3) with significant differences. However, no significant difference was detected in the grading of IVDD for the lower lumbar spine (L3/4, L4/5, and L5/S1). Additionally, no significant differences between the two groups were established in the average DHI. This finding suggests that TAM can promote IVDD, which is more remarkable in the upper lumbar spine [5]. The reason for the lack of an obvious difference in the lower lumbar spine may be that hormones are not the leading factors for IVDD in the lower lumbar spine. The location of the processes occurring during this stage is highly concentrated in the physiological bending part of the spine, a part with high mobility, which is thus susceptible to injury leading to IVDD [28, 29].

Modic et al. defined Modic changes as pathological signal changes in the upper and lower endplates of the vertebral body and the bone marrow. Modic changes were divided into 3 types by MRI signal changes [15]. Özcan-Ekşi EE et al. found that IVDs with Modic changes in the endplate were more prone to degeneration, especially Modic type I changes [30]. Modic changes are closely related to lower back pain and IVDD, but their specific mechanism is still unclear. We evaluated whether Modic changes occurred in the upper and lower endplates of each IVD by MRI and investigated the relationship between TAM use and Modic changes. In this study, Modic changes mainly occurred in the lower lumbar region, similar to the results of the modified Pfirrmann grading scores. There was no significant difference in the occurrence of Modic changes between the two groups, indicating that TAM had no significant effect on Modic changes.

Therefore, further studies on the effects of estrogen and drugs on IVDD, with the exclusion of other influencing factors, are still needed to elucidate all related mechanisms.

This study has some limitations. First, lower back pain is one of the main symptoms of lumbar IVDD. Considering that the spinal metastasis of breast cancer and osteoporosis may cause different degrees of pain, it is difficult to distinguish whether IVDD is the main cause of lower back pain, and thus, pain assessment was not performed in this study, such as visual analogue scale. Second, the sample size of this study is small, and many confounding factors might have affected the results. Therefore, prospective studies with larger sample sizes and longer follow-up periods are needed to comprehensively investigate the effect of TAM on IVDD in the future.

## 5. Conclusion

This study indicates that there might be some positive correlation between TAM use and lumbar IVDD. In particular, the degeneration of L1/2 and L2/3 has shown a correlation with TAM use.

## Figures and Tables

**Table 1:** Modified Pfirrmann grading system.

	Strength of nucleus pulposus and inner annulus fibrosus	Signal difference between the inner and outer sides of the posterior annulus fibrosus	Intervertebral disc height
Grade 1	Homogeneous high signal, equivalent to cerebrospinal fluid (CSF)	Obvious	Normal
Grade 2	High signal, lower than CSF, higher than presacral fat	Obvious	Normal
Grade 3	High signal, lower than presacral fat	Obvious	Normal
Grade 4	Moderate signal, higher than the outer annulus fibrosus	Not obvious	Normal
Grade 5	Low signal, equivalent to low outer annulus fibrosus	Not obvious	Normal
Grade 6	Low signal	Not obvious	Reduced less than 30%
Grade 7	Low signal	Not obvious	Reduced 30%–60%
Grade 8	Low signal	Not obvious	Reduced more than 60%

**Table 2:** Comparisons of patient age, BMI, postoperative history, lumbar lordosis, and average vertebral CT density.

	Age (years)	BMI (kg/m^2^)	Postoperative history (years)	Lumbar lordosis (°)	Average vertebral CT density
TAM group (*n* = 46)	47.43 ± 6.37	24.85 ± 4.83	5.29 ± 3.65	31.15 ± 9.26	185.21 ± 53.66
Non-TAM group (*n* = 29)	46.72 ± 7.14	24.27 ± 5.49	5.31 ± 2.74	30.54 ± 6.96	184.23 ± 50.03
*P* value	0.655	0.635	0.983	0.761	0.937

**Table 3:** Comparisons of the lumbar disc height index for each lumbar disc and the average value.

	Disc height index (%)					
	L1/2	L2/3	L3/4	L4/5	L5/S1	Average
TAM group (*n* = 46)	24.96 ± 4.04	27.28 ± 5.16	30.05 ± 5.70	32.06 ± 5.21	28.63 ± 6.18	29.69 ± 3.08
Non-TAM group (*n* = 29)	26.86 ± 3.41	29.68 ± 3.72	31.23 ± 3.81	31.35 ± 5.11	28.49 ± 7.46	28.52 ± 3.47
*P* value	0.038^*∗*^	0.034^*∗*^	0.522	0.561	0.930	0.141

^*∗*^ indicates a significant difference (*P* < 0.05).

**Table 4:** Comparisons of modified Pfirrmann grading of L1/2-L5/S1 IVD between the TAM group and the non-TAM group.

	TAM group (*n* = 46)/non-TAM group (*n* = 29)				
	L1/2	L2/3	L3/4	L4/5	L5/S1
Grade 1	0/0	0/0	0/0	0/0	0/0
Grade 2	24/25	17/17	6/8	2/3	4/4
Grade 3	12/2	16/10	19/12	10/9	13/3
Grade 4	8/1	8/1	14/9	23/12	11/13
Grade 5	0/1	3/0	5/0	9/3	10/6
Grade 6	1/0	1/1	2/0	1/2	8/3
Grade 7	1/0	1/0	0/0	1/0	0/0
Grade 8	0/0	0/0	0/0	0/0	0/0
*P* value	0.004^*∗*^	0.025^*∗*^	0.052	0.195	0.902

^*∗*^ indicates a significant difference (*P* < 0.05).

**Table 5:** Comparisons regarding the occurrence of Modic changes between the TAM group and the non-TAM group.

	L1/2	L2/3	L3/4	L4/5	L5/S1
TAM group (*n* = 46)	0	0	1	3	4
Non-TAM group (*n* = 29)	0	0	0	1	3
*P* value	—	—	0.427	0.567	0.812

## Data Availability

The data used to support the findings of this study are available from the corresponding author upon request.

## Abbreviations

BMD: Bone mineral density
BMI: Body mass index
CT: Computed tomographic
CSF: Cerebrospinal fluid
ECM: Extracellular matrix
ER: Estrogen receptor
IVD: Intervertebral disc
IVDD: Intervertebral disc degeneration
MRI: Magnetic resonance imaging
NPCs: Nucleus pulposus cells
LL: Lumbar lordosis
DHI: Disc height index
OVX: Ovariectomy
TAM: Tamoxifen.
